# In-hospital outcomes of self-expanding and balloon-expandable transcatheter heart valves in Germany

**DOI:** 10.1007/s00392-021-01928-6

**Published:** 2021-09-21

**Authors:** Peter Stachon, Philip Hehn, Dennis Wolf, Timo Heidt, Vera Oettinger, Manfred Zehender, Christoph Bode, Constantin von zur Mühlen, Klaus Kaier

**Affiliations:** 1 Department of Cardiology and Angiology I, Faculty of Medicine and Medical Center, University of Freiburg, Freiburg, Germany; 2 Center of Big Data Analysis in Cardiology (CeBAC), Heart Center Freiburg University, Freiburg, Germany; 3 Institute of Medical Biometry and Statistics, Faculty of Medicine and Medical Center, University of Freiburg, Stefan-Meier-Str. 26, 79104 Freiburg, Germany

**Keywords:** Interventional cardiology, Transcatheter aortic valve replacement, Valve expansion, Mechanism, Aortic stenosis

## Abstract

**Introduction:**

The effect of valve type on outcomes in transfemoral transcatheter aortic valve replacement (TF-TAVR) has recently been subject of debate. We investigate outcomes of patients treated with balloon-expanding (BE) vs. self-expanding (SE) valves in in a cohort of all these procedures performed in Germany in 2018.

**Methods:**

All patients receiving TF-TAVR with either BE (*N* = 9,882) or SE (*N* = 7,413) valves in Germany in 2018 were identified. In-hospital outcomes were analyzed for the endpoints in-hospital mortality, major bleeding, stroke, acute kidney injury, postoperative delirium, permanent pacemaker implantation, mechanical ventilation > 48 h, length of hospital stay, and reimbursement. Since patients were not randomized to the two treatment options, logistic or linear regression models were used with 22 baseline patient characteristics and center-specific variables as potential confounders. As a sensitivity analysis, the same confounding factors were taken into account using the propensity score methods (inverse probability of treatment weighting).

**Results:**

Baseline characteristics differed substantially, with higher EuroSCORE (*p* < 0.001), age (*p* < 0.001) and rate of female sex (*p* < 0.001) in SE treated patients. After risk adjustment, no marked differences in outcomes were found for in-hospital mortality [risk adjusted odds ratio (aOR) for SE instead of BE 0.94 (96% CI 0.76;1.17), *p* = 0.617] major bleeding [aOR 0.91 (0.73;1.14), *p* = 0.400], stroke [aOR 1.13 (0.88;1.46), *p* = 0.347], acute kidney injury [OR 0.97 (0.85;1.10), *p* = 0.621], postoperative delirium [aOR 1.09 (0.96;1.24), *p* = 0.184], mechanical ventilation > 48 h [aOR 0.98 (0.77;1.25), *p* = 0.893], length of hospital stay (risk adjusted difference in days of hospitalization (SE instead of BE): − 0.05 [− 0.34;0.25], *p* = 0.762) and reimbursement [risk adjusted difference in reimbursement (SE instead of BE): − €72 (− €291;€147), *p* = 0.519)] There is, however, an increased risk of PPI for SE valves (aOR 1.27 [1.15;1.41], *p* < 0.001). Similar results were found after application of propensity score adjustment.

**Conclusions:**

We find broadly equivalent outcomes in contemporary TF-TAVR procedures, regardless of the valve type used. Incidence of major complications is very low for both types of valve.

**Supplementary Information:**

The online version contains supplementary material available at 10.1007/s00392-021-01928-6.

## Introduction

Transcatheter aortic valve replacement (TAVR) as a treatment for aortic stenosis (AS) has only been available for less than 20 years now [1]. The technique has evolved very rapidly over this period and has seen widespread adoption beyond its initial primary indication for use in patients whose risk profile makes surgical valve replacement (SAVR) too dangerous. Current ESC/EACTS Guidelines recommend TAVR via the transfemoral access route (TF-TAVR) even in intermediate-risk patients [2]. Today, TAVR is the most commonly used treatment for AS in Europe [3] and the United States [4].

Two principles of valve expansion were developed from the beginning of the TAVR era and are now well established: expansion via inflation of a balloon (balloon expandable, BE) or self-expansion (SE). The two most widely used valve types are the BE Edwards Sapien and the SE Medtronic Evolut/Corevalve. Both valve types showed convincing results compared to SAVR in randomized controlled trials for high, intermediate and low-risk patients suffering from severe aortic valve stenosis [5–10].

Based on the results of those studies, the two designs are meant to be interchangeable with each other in clinical practice. The parallel availability of two quite different valve expansion methods naturally raises the question of whether or not one of these methods produces superior results. In order to elucidate differences in clinical outcomes, several studies were recently published. The SOLVE-TAVI trial randomized 447 patients in Germany to either SE Evolut R or BE Sapien 3 valve and confirmed equivalent outcomes [11]. However, two retrospective analyses including cases performed in France between 2013 and 2018 found superior outcomes of BE valves in clinical practice [12, 13].

The present study aims to provide further evidence from clinical practice by analyzing results from BE and SE TAVR performed in Germany—a country with a high rate of TAVR procedures. By focusing on the year 2018, the analysis ensures that the use of the most recent valve technologies and the performance of a state-of-the-art procedure are evaluated.

## Methods

### Data

Since 2005, data on all hospitalizations in Germany have been available for scientific use via the Diagnosis Related Groups statistics collected by the Research Data Center of the Federal Bureau of Statistics (DESTATIS). These hospitalization data, including diagnoses and procedures, are a valuable source of representative nationwide data on the in-hospital treatment of patients. This database represents a virtually complete collection of all hospitalizations in German hospitals that are reimbursed according to the Diagnosis Related Groups system. From this database, we extracted data on 17,295 cases of isolated TF-TAVR procedures conducted in 2018. As described previously, patients with a baseline diagnosis of pure aortic regurgitation (main or secondary diagnosis other than I35.0, I35.2, I06.0, I06.2) and those with concomitant cardiac surgery or percutaneous coronary intervention were not included in this analysis [14]. A complete list of procedure codes may be found in Table S1.

Our study did not involve direct access by the investigators to data on individual patients but only access to summary results provided by the Research Data Center. Therefore, approval by an ethics committee and informed consent were determined not to be required, in accordance with German law. All summary results were anonymized by DESTATIS. In practice, this means that any information allowing the drawing of conclusions about a single patient or a specific hospital was censored by DESTATIS to guarantee data protection. Moreover, in order to prevent the possibility to draw conclusions to a single hospital the data are verified and situationally censored by DESTATIS in those cases.

### Endpoints

The analysis focused on eight different end points: in-hospital mortality, bleeding events, stroke, acute kidney injury, postoperative delirium, permanent pacemaker implantation (PPI), mechanical ventilation exceeding 48 h, length of hospital stay and reimbursement. Stroke and acute kidney injury were defined using ICD, Tenth Revision (ICD-10) codes (secondary diagnosis I63* or I64 and N17*, respectively).

Bleeding was defined as requiring a transfusion of > 5 units of red blood cells and identified using OPS-codes (8–800.c1 to 8–800.cr). In-hospital mortality, length of mechanical ventilation, and length of hospital stay were part of DESTATIS’ main set of variables. For all other comorbidities, the existing anamnestic or acute distinctive codes were used (we have discussed OPS and ICD codes in detail previously [14]).

For calculation of the estimated logistic EuroSCORE (European System for Cardiac Operative Risk Evaluation), we were able to populate all fields except for critical preoperative state and left ventricular function. In these, we assumed an inconspicuous state (i.e., no critical preoperative state and no left ventricular dysfunction) and thus calculated a best-case scenario.

### Statistical analysis

In a previous study, Reinöhl et al. [14] identified 20 baseline patient characteristics to describe risk profiles between procedural groups. Since patients were not randomized to the two treatment options (TF-TAVR using either BE or SE valve), logistic or linear regression models were used with these 20 baseline patient characteristics included as potential confounders (all covariates listed in Table 1). Nonelective emergency performance of the procedure and case volume per center per year were also added as confounders. To account for the correlation of error terms of patients treated in the same hospital, a random intercept was included at the center level. See Table S2 for results of the different regression analyses.

**Table 1:** Baseline characteristics of patients with balloon-expandable or self-expanding TAVR procedures in 2018

	Balloon-expandable		Self-expanding		*p* values
*N*	7,413		9,882		
Logistic EuroSCORE^1^ (mean/SD)	12.87	9.67	13.90	9.94	< 0.001
Age in years (mean/SD)	80.48	6.39	81.59	5.79	< 0.001
Female (%)	42.57%		57.14%		< 0.001
NYHA II (%)	13.81%		12.76%		0.043
NYHA III or IV (%)	52.88%		51.59%		0.092
CAD (%)	52.35%		49.84%		0.001
Hypertension (%)	62.12%		64.60%		0.001
Previous MI within 4 months (%)	1.63%		1.57%		0.741
Previous MI within 1 year (%)	0.90%		0.57%		0.009
Previous MI after 1 year (%)	5.05%		3.58%		< 0.001
Previous CABG (%)	8.47%		8.17%		0.471
Previous cardiac surgery (%)	11.95%		13.49%		0.003
Peripheral vascular disease (%)	8.97%		8.19%		0.068
Carotid disease (%)	7.43%		5.88%		< 0.001
COPD (%)	11.45%		11.83%		0.445
Pulmonary hypertension	22.45%		20.04%		< 0.001
Renal disease, GFR < 15% (%)	2.60%		2.08%		0.024
Renal disease, GFR < 30% (%)	3.98%		4.49%		0.098
Atrial fibrillation (%)	44.76%		45.51%		0.328
Diabetes (%)	31.90%		32.16%		0.721
Notfall (%)	9.54%		9.87%		0.470
Number of cases per center (mean/SD)	285.86	137.43	289.02	138.68	0.158

As a sensitivity analysis, potential confounding factors were taken into account using the propensity score methods. In detail, the propensity score was used for adjustment. The propensity score was estimated using a multivariable logistic regression model, with the two treatment options (TF-TAVR using either BE or SE valve) as the dependent variable and all of the baseline characteristics listed in Table 1 as independent variables. Then, propensity score adjustment was applied using the propensity score as continuous covariate. Again, logistic regression models with a random intercept at the center level were conducted.

No imputation for missing values could be conducted due to the absence of codes indicating that data were missing. If the patient’s electronic health record did not include information on a clinical characteristic, it was assumed that that characteristic was not present. Furthermore, no adjustment for multiple testing was carried out. Thus, *p* values may not be interpreted as confirmatory but are descriptive in nature and inferences drawn from the 95% confidence intervals may not be reproducible.

All analyses were performed with Stata 16 (StataCorp, College Station, Texas, USA).

### Results

#### Baseline characteristics

The baseline characteristics of the two patient cohorts are shown in Table 1. SE valves were used somewhat more often, with 7,413 BE procedures and 9,882 SE procedures performed. The cohorts differ significantly in the characteristics EuroSCORE (BE lower), age (BE lower), female sex (more likely in SE), CAD (more likely in BE), hypertension (more likely in SE), previous MI after 1 year (more likely in BE), previous cardiac surgery (more likely in SE), carotid disease (more likely in BE) and pulmonary hypertension (more likely in BE). The difference between groups is particularly marked regarding patient sex, with only 42.57% of patients receiving a BE valve being female, but 57.14% of patients receiving a SE valve. Interestingly, the share of non-elective emergency performance of the procedures and case volumes per center per were highly comparable between the two treatment options.

### Unadjusted in-hospital outcomes

Unadjusted in-hospital outcomes are shown in Table 2. No marked difference was found for the outcomes in-hospital mortality (2.32% BE, 2.21%SE, *p* = 0.617), bleeding (2.35% BE, 2.27% SE, *p* = 0.727) and mechanical ventilation > 48 h (2.00% BE, 2.00% SE, *p* = 0.973). BE valves were associated with a lower risk for stroke (1.70% BE, 2.19% SE, *p* = 0.023), PPI (11.52% BE, 14.33% SE, *p* < 0.001) and postoperative delirium (7.70% BE, 8.93% SE, *p* = 0.004), while SE valves were associated with less acute kidney injury (8.92% BE, 8.03% SE, *p* = 0.039), a shorter length of hospital stay (12.54 days BE, 12.20 days SE, *p* = 0.004) and less reimbursement (€28,846 BE, €28,630 SE, *p* = 0.009).

**Table 2:** In-hospital outcomes of patients with balloon-expandable or self-expanding TAVR procedures in 2018

	Balloon-Expandable		Self-Expanding		*P* value
*N*	7,413		9,882		
In-hospital mortality	2.32%		2.21%		0.617
Bleeding	2.35%		2.27%		0.727
Stroke	1.70%		2.19%		0.023
AKIN	8.92%		8.03%		0.039
Delirium	7.70%		8.93%		0.004
PPI	11.52%		14.33%		< 0.001
Mechanical ventilation > 48 h	2.00%		2.00%		0.973
Length of hospital stay	12.54	8.13	12.20	7.38	0.004
Reimbursement	28,846 €	5,610 €	28,630 €	5,171 €	0.009

### Risk adjusted in-hospital outcomes

**Fig. 1:**
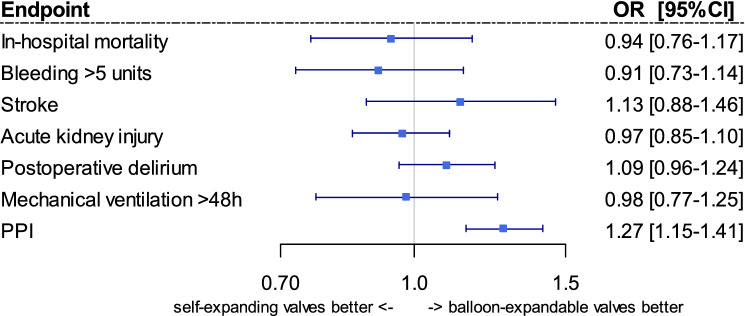
Risk adjusted in-hospital outcomes of patients with balloon-expandable or self-expanding TAVR procedures in 2018

Adjusted results are shown in Fig. 1. No marked difference in outcomes was found for in-hospital mortality (risk adjusted odds ratio (aOR) for SE instead of BE 0.94 [96%CI 0.76;1.17], *p* = 0.617), major bleeding (aOR 0.91 [0.73; 1.14, *p* = 0.400), stroke (aOR 1.13 [0.88; 1.46], *p* = 0.347), acute kidney injury (aOR 0.97 [0.85; 1.10], *p* = 0.621), postoperative delirium (aOR 1.09 [0.96; 1.24], *p* = 0.184), mechanical ventilation > 48 h (aOR 0.98 [0.77;1.25], *p* = 0.893), length of hospital stay (risk adjusted difference in days of hospitalization: − 0.05 [− 0.34;0.25], *p* = 0.762) and reimbursement (− €72 [-€291;€147], *p* = 0.519). There is, however, an increased risk of PPI for SE valves (aOR 1.27 [1.15;1.41], *p* < 0.001).

See Table S2 for results of the different regression analyses.

Similar results were found after the propensity score approach. Detailed results of the propensity score approach can be found in Table S2.

## Discussion

In our analysis of in-hospital outcomes in a large national cohort of more than 17,000 patients with aortic stenosis treated with TAVI, no systematic difference was observable between balloon and self-expandable TAVI valves.

The risk for in-hospital mortality, major bleeding, acute kidney injury, postoperative delirium, prolonged mechanical ventilation, or prolonged hospital stay was similar between BE and SE TAVI valves in Germany in 2018. Our results offer a logical comparison with two registries reported from a recent French national registry by Van Belle et al. [12] and a study based on French administrative data by Deharo et al. [13], although Deharo et al. do not report in-hospital outcomes.

The German and Van Belle cohorts are comparable with regard to age and risk profile such as measured by the EuroSCORE, with the French cohort being slightly older and having a slightly higher EuroSCORE. The Deharo cohort is slightly older, with no EuroSCORE given. In total more TAVR procedures are performed in Germany, with over 17,000 cases in 2018 compared to about 6,000 cases per year (31, 113 between 2014 and 2018) in France, as reported by Deharo et al. When regarding mortality as primary endpoint, the difference is relvant. In the Van Belle cohort, with patients recruited from 2013–2015, there is an in-hospital mortality of 4.2% for BE and 5.6% for SE valves, whereas the German patients treated in 2018 show mortalities of 2.32% for BE and 2.21% for SE. A previous paper of ours^3^ finds an in-hospital mortality for TF-TAVR in Germany for 2014 and 2015 of only 3.23%, indicating that the difference is not entirely due to the technological progress in the meantime. A higher experience in Germany may be one reason for the observed differences. This is line with a recent randomized trial with 447 patients^11^ treated in Germany with a 30-day mortality between 2.3 and 3.2%. This study randomized patients to BE and SE valves and found equivalent results for the primary valve-related composite efficacy endpoint. However, in-hospital mortality – a component of the composite endpoint—was significantly lower in the BE treated group.

Two studies analyzing early generation BE or SE valves found comparable outcomes. The randomized CHOICE trial including 241 patients [15] recently published 5-year outcomes, observing no significant difference in outcomes between the valve types, albeit with limited statistical power. Another study from 2016, describing the outcomes of 793 propensity-score matched patients from four European heart centers treated with either Medtronic CoreValve or Edwards SAPIEN/SAPIEN XT, likewise found no differences in outcomes at 30 days and 1 year [16].

The CENTER trial [17] pooled and analyzed data from 10 registries or clinical trials and did find differences in outcomes between the valve types. Incidences of stroke and pacemaker implantation were higher in SE valves, however, incidence of major bleeding was higher in BE valves. Patients receiving SE valves were more likely to suffer from stroke and postoperative delirium, although the risk was not significantly higher after risk adjustment. However, the increase in neurological complications matches the, albeit nonsignificant, findings of Van Belle et al. for stroke and those of the CENTER trial in patients receiving SE valves. Given these findings, further studies addressing potential underlying factors be warranted.

Recent data suggest that valve selection should be adjusted to certain patients’ anatomies, or when particular characteristics are present [18, 19]^1^. In our study and some more recent publications [18, 20], which left the choice of device to the discretion of the heart team, women were significantly more likely to be treated with SE valves, indicating a significant degree of differentiation in target populations for the respective valve types already existing in clinical practice. The reason for the higher use of SE valves might be suprannular engineering, which allows larger TAVR valve areas in smaller annuli.

However, our study suggests that all comers can be treated with any type of valve—whenever anatomically possible—and can expect an excellent procedural outcome.

## Limitations

One limitation of this study is the lack of long-term follow-up, which is due to the fact that the data source used does not include identifying information on individuals, meaning that later hospitalizations or their absence cannot be connected to the patients from our cohort. Our study thus solely provides data on in-hospital outcomes, albeit for a very large, complete national yearly cohort of procedures. Moreover, the administrative data set lacks relevant clinical information (such as echocardiographic findings or anatomical characteristics), preventing operative risk assessment and a better understanding of the underlying valvular pathomechanism. Therefore, only an approximation of the logistic EuroSCORE, in fact a conservative or ‘best-case scenario’ estimate, is applied.

Another limitation is the lack of information in the dataset on the exact model of device used beyond the type—for SE valves, for example, the ACURATE neo [21] or the CoreValve [19] are in use in Germany.

## Conclusion

We find comparable outcomes in contemporary TAVR procedures in Germany regardless of the valve type used. Incidence of major complications is very low for both types of valves, and has further improved from the already low level described during the last years. Our finding of a higher incidence of neurological complications when using self-expanding valves matches that of a comparable previous national cohort from France. Investigation into the causative factors might be warranted if this trend continues to be observed.

## Supplementary Information

Below is the link to the electronic supplementary material.

Supplementary file1 (DOC 34 kb)
