# Supplementary material for: In-hospital outcomes of self-expanding and balloon-expandable transcatheter heart valves in Germany
Source: Clin Res Cardiol. 2021 Sep 21;110(12):1977–82. doi: 10.1007/s00392-021-01928-6 (PMC8639556; doi:10.1007/s00392-021-01928-6)
Supplement: Supplementary file 1 — Supplementary file1 (DOC 34 kb) [file 392_2021_1928_MOESM1_ESM.doc]

**Table S1: Diagnosis and procedure codes used for this analysis**

**Proceduress**

5-35a.03 Transcatheter aortic valve replacement using balloon-expanding valves

5-35a.04 Transcatheter aortic valve replacement using self-expanding valves

5-351.0* Surgical aortic valve replacement

5-361.*, 5-362.*, 5- Coronary artery bypass graft

363.*,

5-351.1*, 5-351.2*, Surgical mitral valve replacement/reconstruction

5-353.1, 5-353.2

5-351.4* Surgical tricuspid valve replacement

5-377.0 et seqq. Permanent pacemaker implantation

| 8-800.7* | Transfusion of RBC |
| --- | --- |
| since 2010: |  |
| 8-800.c* |  |
| **Diagnosis** |  |
| I35.0, I06.0 | Aortic valve stenosis (degenerative/rheumatic) |
| I35.2, I06.2 | Combined aortic valve diseases (degenerative/rheumatic) |
| I50.1* | Left ventricular congestive heart failure *(according to NYHA classes)* |
| I10* | Arterial Hypertension |
| I25.11, I25.12, | Coronary artery disease |
| I25.13 |  |
| I25.20, I25.21, | Previous myocardial infarction *(within 4 months/1 year/after 1 year)* |
| I25.22 |  |
| Z95.1 | Previous coronary artery bypass graft |
| Z95.1 – Z95.4 | Previous cardiac surgery |
| I70.20-I70.25, | Peripheral vascular disease |
| I70.8, I70.9, I73.9 |  |
| I65.2 | Carotid disease |
| I21* | Acute myocardial infarction *(within the last 28 days)* |
| J44* | Chronic obstructive pulmonary disease |
| I27* | Pulmonary hypertension |
| N18* | Renal disease |
| N17* | Acute kidney injury |
| I48.1* | Atrial fibrillation |
| E10* - E14* | Diabetes |
| I63*, I64 | Stroke or cerebral infarction incl. occlusion and stenosis of cerebral and precerebral arteries, resulting in cerebral |
|  | infarction |
